# A Novel Regulator of Activation-Induced Cytidine Deaminase/APOBECs in Immunity and Cancer: Schrödinger’s CATalytic Pocket

**DOI:** 10.3389/fimmu.2017.00351

**Published:** 2017-04-06

**Authors:** Justin J. King, Mani Larijani

**Affiliations:** ^1^Immunology and Infectious Diseases Program, Division of Biomedical Sciences, Faculty of Medicine, Memorial University of Newfoundland, St. John’s, NL, Canada

**Keywords:** lymphoma, antibodies, DNA mutations, leukocytes, enzymes and coenzymes, HIV infections

## Abstract

Activation-induced cytidine deaminase (AID) and its relative APOBEC3 cytidine deaminases boost immune response by mutating immune or viral genes. Because of their genome-mutating activities, AID/APOBECs are also drivers of tumorigenesis. Due to highly charged surfaces, extensive non-specific protein–protein/nucleic acid interactions, formation of polydisperse oligomers, and general insolubility, structure elucidation of these proteins by X-ray crystallography and NMR has been challenging. Hence, almost all available AID/APOBEC structures are of mutated and/or truncated versions. In 2015, we reported a functional structure for AID using a combined computational–biochemical approach. In so doing, we described a new regulatory mechanism that is a first for human DNA/RNA-editing enzymes. This mechanism involves dynamic closure of the catalytic pocket. Subsequent X-ray and NMR studies confirmed our discovery by showing that other APOBEC3s also close their catalytic pockets. Here, we highlight catalytic pocket closure as an emerging and important regulatory mechanism of AID/APOBEC3s. We focus on three sub-topics: first, we propose that variable pocket closure rates across AID/APOBEC3s underlie differential activity in immunity and cancer and review supporting evidence. Second, we discuss dynamic pocket closure as an ever-present internal regulator, in contrast to other proposed regulatory mechanisms that involve extrinsic binding partners. Third, we compare the merits of classical approaches of X-ray and NMR, with that of emerging computational–biochemical approaches, for structural elucidation specifically for AID/APOBEC3s.

## Importance and Challenges of Solving AID/APOBEC Structures

Activation-induced cytidine deaminase (AID) is a 198 amino acid DNA-editing enzyme that deaminates deoxycytidine (dC) to deoxyuridine (dU) in single-stranded DNA (ssDNA) (1–6). It acts on immunoglobulin (Ig) loci initiating hypermutation and recombination events that lead to improved and class-switched antibodies (1, 7–9). However, AID is loosely targeted to Ig loci, and hence, it induces genome-wide mutations and double-strand breaks which can lead to tumors (10–16). In addition, continued AID expression increases genetic plasticity of tumors thereby accelerating disease progression (17, 18).

Activation-induced cytidine deaminase is a member of the apolipoprotein B RNA-editing catalytic component (AID/APOBEC) family of cytidine deaminases, a Zn-dependent family with 11 members in humans: AID, APOBEC1, APOBEC2, the APOBEC3 sub-branch (A-H, excluding E), and APOBEC4 (9, 19, 20). The APOBEC3 (A3) sub-branch members are anti-retroviral/retroelement restriction factors thereby also playing an immune function (21, 22); however, in the last few years, a role in cancer initiation has also emerged for the A3 sub-branch of the family, in particular, A3A, A3B (23–32), and more recently A3H haplotype I (33).

**Figure 1:**
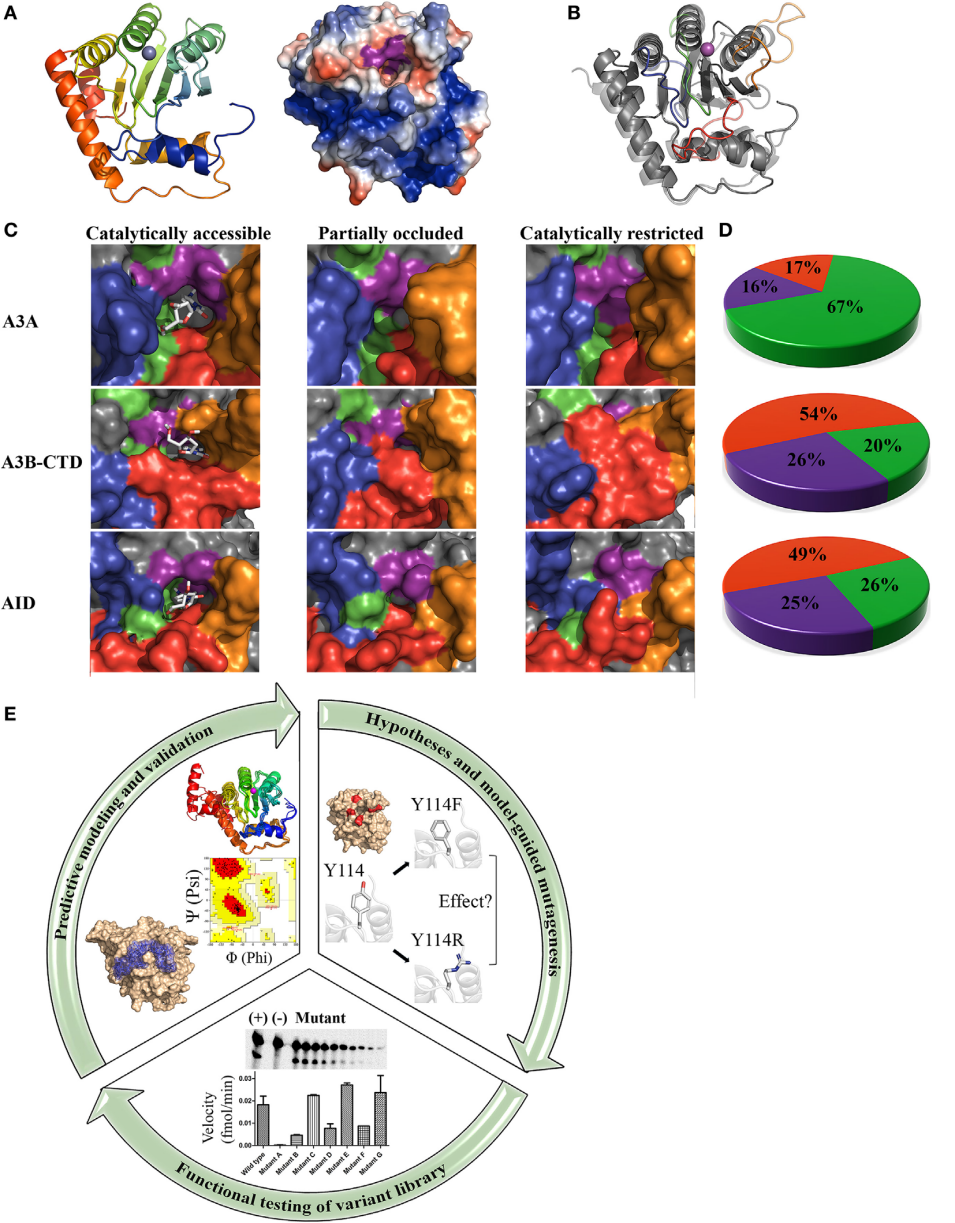
**Core architecture, catalytic pocket occlusion, and computational/biochemical approaches to solving activation-induced cytidine deaminase (AID)/APOBEC structures**. **(A)** Representative ribbon structure (left) and surface topology (right) of AID modeled from APOBEC templates. In the ribbon structure, N- to C-termini progression is shown from blue to red and the gray sphere depicts active site zinc. In the surface topology, positive, negative, and neutral residues have blue, red, and white surfaces, respectively. The Zn-coordinating residues and catalytic glutamic acid surface are colored purple. A distinct feature of AID among the APOBECs is its high positive charge at neutral pH, concentrated along two single-stranded DNA (ssDNA)-binding grooves that pass over the catalytic pocket. **(B)** Ribbon structures of A3A (transparent) and AID (non-transparent) were superimposed. In each protein structure, the secondary catalytic loops 2, 4, 6, and 8 are colored red, orange, green, and blue, respectively. **(C)** Catalytically accessible (left), partially occluded (middle), and catalytically restricted (right) conformations of A3A (top), A3B-CTD (center), and AID (bottom). The surface of secondary catalytic loop 2, 4, 6, and 8 were colored red, orange, green, and blue, respectively, to correspond with the ribbon structure shown in panel B. Catalytically accessible conformations are shown with bound dC in the catalytic pocket. Conformations were deemed catalytically accessible if they bound dC in a deamination-feasible configuration in the catalytic pocket *via* molecular docking [AutoDock VINA (59)]. In catalytically restricted conformations, the secondary catalytic loops adopt a configuration that block the pocket. **(D)** Proportion of catalytically restricted (red), partially occluded (purple), and catalytically accessible (green) conformations in A3A (top), A3B-CTD (center), and AID (bottom). A3A showed a dramatically higher proportion of catalytically accessible conformations in comparison to A3B-CTD and AID. NMR conformations of A3A (PDB: 2M65), A3B (PDB: 2NBQ), and previously reported structures of AID (58) were used. **(E)** Combinatorial computational/biochemical approach for solution of functional and native enzyme structures. A library of thousands of predicted structures is generated through homology modeling with a range of suitable template structures, generating multiple low energy conformations. The resulting conformational ensemble is then evaluated mathematically (e.g., Ramachandran and other means of evaluating model quality). Models are also checked for concordance with known biochemical properties of the enzyme. Molecular docking can be used to determine the substrate binding regions of the active site and surrounding regions. Concurrently, specific hypotheses are formed based on the highest confidence predicted conformations and their interaction with substrate. To test these hypotheses and to validate the positions and relative attitudes of specific core or surface residues, a large variant library ought to be constructed and tested in functional enzyme assays. This library can include point mutants, multiple mutants, orthologous and chimeric versions of the enzyme. For key residues involved in catalysis regulation, several point mutations spanning the range of synonymous to severe are more informative. Functional evaluation of this variant library ought to be used to confirm the involvement of key residues/motifs in specific biochemical aspects such as substrate binding, catalysis, and structural stability. Collectively, information from functional testing of the variant library is used to refine and validate the predicted enzyme structure and its interactions with substrate, to yield a functional and native structure.

Given their intimate links to immunity and cancer, much effort has been placed on understanding the molecular structures of AID/APOBECs over the last decade. A major hurdle in this effort has been the isolation and purification of native AID/APBOEC proteins to absolute purity. To different measures for each individual family member, the challenges include cellular genotoxicity, highly charged surfaces mediating extensive non-specific protein–protein, protein–DNA/RNA interactions (9, 34), and polydisperse oligomerization (35, 36). Consequently, in most cases, structure resolution by the traditional methodologies of X-ray crystallography and NMR has necessitated substantial alterations to stabilize protein charge and enhance solubility or crystallization. Therefore, the vast majority (22 of 24) of APOBEC structures solved by X-ray or NMR are of truncated and/or mutated versions, necessary to enhance solubility (Table 1) (37–57). Nevertheless, it has become clear that AID/APOBEC family member enzymes share the core structure of a central β-sheet with 4 or 5 β strands sandwiched between 6 and 7 α-helices, connected by 12–13 flexible loops of variable lengths (Figure 1A). Second, they all have highly charged DNA-binding grooves, necessary to bind negatively charged polynucleotides. The arrangement of core catalytic residues in the catalytic pocket is also conserved, consisting of a Zn-coordinating triad of two cysteines and a histidine, atop a catalytic proton-donor glutamic acid (C87, C90, H56, and E58 in AID) (9, 19, 20, 58).

**Table 1:** **All X-ray and NMR solution structures of the APOBEC family**.

APOBEC	Experimental method	Truncations	Mutations	PDB ID
Hs-A2	X-ray	Truncated (Δ1–40)	N/A	2NYT
Mouse-A2	NMR solution	Truncated (Δ1–45)	N/A	2RPZ
Hs-A3A	NMR solution	N/A	N/A	2M65
Hs-A3A	X-ray	N/A	E72A, C171A	4XXO
Hs-A3A	X-ray	Truncated (Δ196–199)	E72A	5SWW
Hs-A3B	X-ray	Truncated (Δ1–186, Δ242–248)	F200S, W228S, L230K, Y250S, F308K	5CQK
Hs-A3B	NMR solution	Truncated (Δ1–186)	N/A	2NBQ
Hs-A3B	X-ray	Truncated (Δ1–186, Δ205–207, Δ242–249)	F200S, V205G, L209I, R210G, R212H, Q213K, W228S, L230K, Y250S, E255A, F308K	5TD5
Hs-A3C	X-ray	N/A	N/A	3VOW
Hs-A3F	X-ray	Truncated (Δ1–184)	Y196D, H247G, C248R, C259A, F302K, W310D, Y314A, Q315A, K355D, K358D, F363D	4IOU
Hs-A3F	X-ray	Truncated (Δ1–217)	N/A	4J4J
Hs-A3F	X-ray	Truncated (Δ1–186)	N/A	3WUS
Hs-A3F	X-ray	Truncated (Δ1–184)	Y196D, H247G, C248R, C259A, F302K, W310D, K355D, K358D, F363D	5HX5
Hs-A3F (Zn-depleted)	X-ray	Truncated (Δ1–184)	Y196D, H247G, C248R, C259A, F302K, W310D, K355D, K358D, F363D	5HX4
Hs-A3G	NMR solution	Truncated (Δ1–197)	L234K, C243A, F310K, C321A, C356A	2JYW
Hs-A3G	X-ray	Truncated (Δ1–196)	N/A	3E1U
Hs-A3G	NMR solution	Truncated (Δ1–192)	N/A	2KBO
Hs-A3G	NMR solution	Truncated (Δ1–190)	L234K, C243A, F310K, C321A, C356A	2KEM
Hs-A3G	X-ray	Truncated (Δ1–194)	L234K, C243A, F310K, C321A, C356A	3IR2
Hs-A3G	X-ray	Truncated (Δ1–194)	L234K, C243A, F310K, C356A	3V4K
Hs-A3G	X-ray	Truncated (Δ1–192)	D370A	4ROW
Hs-A3G	NMR	Truncated (Δ1–11, Δ78, Δ143–146, Δ197–384)	Y13D, R14P, Y22N, L62D, F71L, H72S, W73L, F74V, T101A, A109Q, D110P, P111T, K112H, F126A, C139A, K141A, R142G, M149I, R169G, E170A, L171P, E173Q, N176D, N177G, P179D, K180E, Y181H, Y182S, I183Q, L184A, H186S, I187G, M189R	2MZZ
Primate-A3G	X-ray	Truncated (Δ139–146, Δ197–384)	(C139-Q140-K141-R142-D143-G144-P145-H146) replaced with (A-E-A-G) residues	5K83
Hs-AID	X-ray	Truncated (Δ1–4, Δ20–22, Δ184–198)	N7D, R8P, R9H, K10I, L12T, Y13S, Q14N, K16N, V18G, R19I, R25H, E26K, V32E, K34E, R36L	5JJ4

*Most APOBEC structures are heavily modified through mutations and/or truncations. Δ denotes the amino acids that were deleted from the structure. In cases where a study has reported several protein databank IDs of highly similar structures, a representative PDB code is listed*.

## Solving the Structure of AID and Discovery of Catalytic Pocket Closure in AID/APOBECs

The biochemical properties of AID have been previously described. AID has an exceptionally high affinity (nM range) for binding ssDNA and an unusually slow catalytic rate of one reaction in several minutes (5, 60), ~2,000 times slower than a typical enzyme (61). We postulated that this catalytic lethargy and high-binding affinity to DNA had evolved to protect genomes from rampant AID activity (9). In direct support of this notion, mutants of AID with higher catalytic rates were shown to mediate higher levels of genome damage in cells (62). Although this body of work led to understanding AID’s behavior, the molecular basis behind these properties remained an enigma. AID is notoriously challenging to isolate to absolute purity and hence its native structure has remained unsolved by X-ray and NMR since its discovery in 1999, despite intense efforts. Thus, we posited that even if AID’s structure were to be solved by traditional methods of X-ray or NMR, it would most likely be of a truncated and/or heavily mutated version. We proposed an alternative methodology to gain insight into the functional and native structure of AID. We utilized eight recently solved structures of AID’s APOBEC relatives as templates to generate thousands of AID predicted model structures followed by identification of the lowest energy clusters (58) (Figure 1A). Concurrently and guided by the computational predictions, we generated a library of 400 AID variants and carried out extensive biochemical characterization of catalytic function and DNA binding to rigorously test key predictions of models. This library included different point mutants for each residue along the length of AID, orthologous AIDs, and chimeras involving regions of other deaminases exchanged into the AID scaffold, or vice-versa. Our rationale for including AID orthologs was that divergent AID from distantly evolved species may have distinct biochemical properties and characterizing these through a combination of homology structural modeling and functional analysis of mutated and chimeric enzymes would generate structure:function insights. Differences among orthologous AIDs included catalytic rates, substrate preferences, DNA-binding affinities, and thermosensitivity profiles (63–65). Since these differences are typically due to structural features, being reflective of catalytic motifs, surface composition, and overall protein flexibility, respectively, characterizing the basis of these differences among orthologs proved a valuable tool to gain insight into AID’s structure:function relationships. This computational–biochemical approach led to the first relatively detailed 3D maps of AID’s functional structure with special focus on catalytic pocket architecture and ssDNA-binding motifs (9, 58, 63, 64).

The architecture and dynamics of an enzyme’s catalytic pocket are important determinants of its activity. In addition to the core catalytic pocket composed of the aforementioned triad Zinc-coordinating residues and a Glutamic acid, we identified an additional 21 amino acids that are not directly involved in the deamination reaction, but compose the pocket’s physiochemical microenvironment (58). These residues termed secondary catalytic residues form the “walls” and “floors” of the pocket and stabilize dC binding. We noted that the conformations of these secondary catalytic residues exhibited more variability than that of the primary catalytic residues among predictions, because these residues reside on several highly flexible connecting loops without secondary structures of their own, that surround the catalytic pocket (Figure 1B). Because of this placement, we observed that the catalytic pocket of AID appeared to be only marginally stable such that ~75% of conformations exhibit a occluded pocket unable to accommodate dC. Thus, we hypothesized that dynamic catalytic pocket closure is a built-in mechanism that limits AID activity.

There are several lines of indirect but strong evidence for the existence of catalytic pocket closure in AID/APOBEC3s. First, the fact that the majority of AID conformations exist in a state with closed catalytic pockets provides a mechanistic explanation for the relative catalytic lethargy of AID as discussed above (5). Second, it is mathematically compatible with known parameters of AID: AID binds ssDNA sporadically on its surface such that most ssDNA (~95%) neither pass over AID’s catalytic pocket, nor position dC for catalytic pocket entry. The proportion of catalytically viable AID:DNA complexes (~5%) multiplied by the ratio of open pockets (25%) yields (1.25%). This correlates closely with our own estimates of active AID complexes based on Michaelis–Menten parameters (5) and with other studies that carried out mathematical modeling of AID’s substrate catalysis (66).

Direct proof for existence and significance of catalytic pocket closure came from two sources: one functional, and the other structure-based. First, we designed a panel of AID variants in which the secondary catalytic loops and surrounding regions were replaced with their equivalents from other APOBECs or orthologous AIDs to alter predicted pocket dynamics such that the pocket would spend either more or less time in the open conformation. We then observed that the proportion of time the pocket was predicted to assume an open conformation correlated exquisitely with catalytic rate differences among said AID variants some of which became up to 100 times more active than wild-type AID because of a catalytic pocket that spends more time in the open conformation. This provided direct functional evidence that pocket closure limits activity. The second proof came from direct observation of closed pockets in several siblings of AID: in APOBEC3A by NMR (40), in APOBEC3B by X-ray crystallography (42, 57), and by NMR (43) (Figures 1C,D).

## Differential Catalytic Pocket States Mediate Variable Biological Activities among AID/APOBECs

From an evolutionary perspective, regulation by catalytic pocket closure provides an effective means to fine-tune variable levels of enzymatic robustness across the AID/APOBEC family, as well as impart varying types of activities among orthologous versions of each family member. This is because the same high degree of movement freedom in the secondary catalytic loops that lead to the fluidity of catalytic pocket dynamics in each AID/APOBEC3 enzyme also allows for a high level of sequence and length divergence in these loops among individual AID/APOBEC3s, to impart a unique range of open/closed breathing dynamics to the catalytic pocket of each member (Figure 1).

To elaborate, in each APOBEC3, the catalytic pocket “walls” and “floor” are composed of residues contributed by four secondary catalytic loops (Figure 1B). The highest structural variation among the AID/APOBEC family appears in loop 2 (L2), loop 4 (L4), and loop 8 (L8) with respect to sequence homology, length, and compaction relative to the core enzyme structure (Figures 1B,C) (58). L2 contains residues involved in ssDNA-binding, catalytic pocket and dC stabilization, substrate specificity, and 5-mC tolerance (58, 67–69). L4 contains residues critical to catalysis and catalytic pocket occlusion (58). Recently, an allosteric regulatory role for L4 in A3A and A3G was identified through coordination of a secondary Zn that enhances activity (70). Interestingly, secondary Zn coordination was suggested to fine tune the position of the secondary catalytic residues, thus creating an ideal environment for cytidine deamination (70). Furthermore, secondary Zn coordination was suggested to mediate cooperative dimerization. Lastly, L8 mediates substrate sequence specificity, dC stabilization, and 5-methyl-C (5-mC) tolerance (58, 64, 67, 71). Collectively, the secondary catalytic loops mediate functional differences among the AID/APOBECs and dictate variations in the frequency of open vs. closed catalytic pockets.

Based on these observations, we proposed that differences in secondary catalytic loops mediate variable catalytic pocket breathing dynamics, responsible for different enzymatic robustness among AID/APOBEC enzymes (58). Indeed, in the last year, functional evidence in support of this novel mode of regulation has emerged. First, A3A exhibits open catalytic pockets in more conformations than AID (67 vs. 25.6%), and accordingly it is a more robust enzyme with a faster on/off rate of deaminating DNA (40, 72). Second, A3B-CTD exhibits roughly one third of the pockets in an open conformation compared to A3A (20 vs. 67%, respectively) (Figure 1D) and this also correlates directly with a lower catalytic rate (43). It is intriguing that thus far, catalytic pocket occlusion has been observed in three of the most mutagenic and tumorigenic members of the AID/APOBEC family: AID, A3A, and A3B. This lends credence to the idea that this is an internal protective mechanism to limit genome mutations by these enzymes. As mentioned above, the difference in catalytic activity of purified AID, A3A, A3B-CTD, correlates with the ranking of pocket occlusion (Figure 1D). Though further study is required to clarify the relative contributions of A3A, A3B, and other APOBEC3 branch enzymes (e.g., A3H) in various types of cancers, some emerging evidence indicates that there is a more dominant mutational signature observed from A3A than A3B, at least in a yeast model and in urothelial carcinoma, despite lower levels of A3A expression (28, 73).

In addition to regulation of tumorigenic activity, differences in catalytic pocket dynamics also appear to correlate well with other biological functions of AID/APOBECS. As an example, zebrafish AID has a significantly higher reaction rate than human AID and is also unique among all AID orthologs in that it can deaminate 5-mC in methylated CpG motifs (64). This explains a puzzling previous report that zebrafish AID plays a completely non-immune role. During embryogenesis in zebrafish, AID can mediate promotor demethylation through erasure of gene-silencing CpG methylation marks, thus orchestrating widespread gene expression required for tissue differentiation (74). This is attributable to conformational differences in the aforementioned secondary catalytic loops between human and zebrafish AID, which translate to a higher ratio of open vs. closed catalytic pockets. Consequently, zebrafish AID can accommodate and deaminate 5-mC, as opposed to human AID whose activity on 5-mC is negligible. This enzymatic difference is one factor that enables zebrafish AID to function in genome demethylation during embryonic development, an activity that is completely outside the realm of an immune function (58, 64). Taken together, these lines of evidence are supportive of catalytic pocket occlusion being a key regulator of biological functions of AID/APOBEC3s, including their role in tumorigenic genome damage.

## Catalytic Pocket Occlusion as Internally Built-in Regulation

Since the discovery of AID, much effort has been directed at understanding how its activity is regulated, under the supposition that a mutator so threatening must be operating under tight restrictions. To date, almost all efforts have focused on modes of regulation that are extrinsic to the enzyme itself. This has led to the identification of over two dozen co-factors proposed to bind AID either directly or indirectly through associations with other proteins or DNA/RNA (75–99). The list of putative binding partners is rather large for a relatively small protein of 198 residues. Hence, one must approach biological relevance with caution for several reasons: first, although some co-factors are modestly enriched at Ig loci, none can account for targeting AID to specific loci. Second, given the relatively small size of AID and the lack of clear conformational protein-binding domains, the number of proposed co-factors seems high. It is rather improbable that a small 198 aa globular enzyme can fold properly to bind ssDNA, deaminate dC, maintain sequence specificity, while still leaving enough non-essential portions free to bind dozens of different co-factors each in a specific and orchestrated fashion. Indeed, a careful analysis of AID’s structure reveals that most of its structure can be ascribed a function directly related to forming the core architecture essential to bind and deaminate a polynucleotide. Furthermore, a portion of the surface is likely unavailable due to forming the oligomerization surfaces, as most AID/APOBECs appear to exist as dimers or tetramers (5, 38, 70, 100). Also, AID has a highly charged surface and a well-known propensity for high affinity non-specific interactions with other proteins (9, 34). Thus, the biological significance of AID binding to many of its putative co-factors is a topic that requires further resolution. Furthermore, the very premise of searching for cofactors to explain regulation of AID targeting may be flawed in that the more AID is studied, the clearer it becomes that its activity is rather not tightly regulated: despite a modest preference for Ig loci which appears to be mediated by unique transcriptional features (101–103), AID mutates endogenous genes and transgenes genome-wide, and can do so in any cell type in which it is naturally or exogenously expressed (10, 104–107).

Like AID, the search for regulatory mechanisms of other APOBEC3s has also focused on extrinsic binding factors, of which several have been identified including various viral proteins, and transcription factors (108–111); the most well-characterized APOBEC3 are virion infectivity factor (Vif) and cytoplasmic ribonuclear complexes. The Vif protein of HIV binds and targets A3C, A3G, A3F, A3D, and A3H (to varying degrees) for degradation *via* a ubiquitin-dependent proteosomal pathway (44, 112–120). Thus, when Vif is present, APOBEC3 effectiveness in viral restriction is severely diminished. Second, the activity of the anti-retroviral APOBEC3s is limited by entrapment in high-molecular-mass ribonuclear complexes (HMM) that may reach megadaltons in size, mediated by non-specific protein/DNA/RNA binding in the cytoplasm, mediated by aforementioned highly charged surfaces (121–127).

In contrast to regulation by extrinsic binding partners, be they protein or nucleic acid, catalytic pocket closure represents a novel intrinsic mode of regulation. This simple mechanism of limiting activity has several attractive features: it is ever-present, biologically reliable, mechanistically simple, and structurally sound. Furthermore, as discussed in the preceding section, its variation is an evolutionary efficient mechanism for diversifying and fine-tuning activity levels of family member enzymes, as catalytic pocket closure rates can be adjusted by minimal amino acid substitutions in secondary catalytic loops. It is also biologically efficient since it does not require any cellular resources, unlike the proposed complex networks of co-factors, which themselves would require regulation in different cells at different stages of differentiation or viral infection, thus amplifying the need for cellular resources.

## Importance of Determining AID/APOBEC3 Structures That are Native and Include Functional Insights

X-ray crystallography and NMR have advanced the AID/APOBEC field with the full or partial structure elucidation of 7 of 11 APOBEC enzymes. Despite these achievements, there are pitfalls in using these traditional methods alone. First, the purification issues discussed above have necessitated working with significantly truncated and/or heavily mutated versions of AID/APOBEC proteins (Table 1). The truncations and mutations are often in functionally critical regions, such as the secondary catalytic loops. Additionally, all double-domain APOBECs whose structure has been characterized (A3B, A3F, and A3G) lack their enzymatically inactive N-terminal half which is implicated in the catalytic activity and dimerization (56, 128, 129). The N-terminal half of A3G’s separate structure was recently reported; however, these were also mutated and likewise lack the C-terminal half (55, 56). Second, depending on crystallization or NMR conditions, even the same APOBEC structure determined by different groups can be quite distinct (49, 50). These differences are likely due to differences in solution or crystallization conditions which can bias toward a specific structure or conformation (130).

In contrast, the methodology that we applied to solving AID’s structure provides both a functional and native structure (58). By integrating dynamic modeling with the study of a large library of variants to functionally verify key model predictions, the emerging picture integrates the relative abundance of an enzyme’s conformations with functional significance (Figure 1E). This approach is particularly advantageous in the case of AID/APOBEC3s because many functional differences among AID/APOBEC3 family members appear to be dictated by subtle differences in breathing dynamics, rather than major architectural differences. It is important to note that despite being a robust methodology for determining functional and native enzyme structures, this approach is not without practical challenges: first, it is laborious and time-intensive since it requires examination of thousands of high confidence models. As modeling efforts progress, there is a continuing need to generate and test a large variant library, often necessitating several mutants of each key residue to rigorously verify its exact position, relative attitude, and role(s). In addition, a sensitive enzyme assay able to detect even small differences in biochemical properties with that of wild-type ought to be in place. Practical difficulties are compounded by the fact that this approach of solving a functional and native structure is often most useful for enzymes that are challenging to purify. Second, modeling efforts depend critically on the availability of solved X-ray or NMR structures to serve as templates, with multiple templates increasing confidence. For instance, at the time of our efforts on AID, we utilized eight available APOBEC structures as templates. It is important to have numerous templates from different family members, so as to at least partially compensate for aforementioned limitations of each template in terms of encompassing the full range of conformations. Furthermore, template structures ought to be evaluated for their suitability on the basis of extent and location of homologous/identical residues, and model quality itself ought to be rigorously scrutinized, mathematically and functionally using the variant library (58) (Figure 1E). Third, the basic biochemical properties of the enzyme ought to have previously been determined so as to serve as a valuable verification tool for model validity; since we had already determined that AID has an unusually low catalytic rate and high binding affinity for ssDNA, the fact that our structure fully explained both of these properties through the abundance of closed pockets and positively charged surface residues along putative DNA-binding grooves and elsewhere on the surface, provided further confidence. Lastly, definite physical confirmation of findings requires subsequent observation by X-ray and NMR, as in the case of catalytic pocket closure described above. In addition to our observation of catalytic pocket closure being confirmed by direct X-ray and NMR studies as described above (42, 43, 57), other X-ray studies have also confirmed our observation of key catalytic residues as well as important DNA-binding residues of AID: following the publication of AID’s functional structure, the crystal structure of an AID variant was also reported (37). As expected, it included mutations and truncations crucial to solubilize AID for X-ray crystallography (Table 1). Although this structure represents a significant achievement, it necessitated introducing mutations and truncations that removed some of the unique characteristics of AID. For instance, the majority of mutations neutralized the positively charged surface residues lining the DNA-binding groove culminating in a net charge of +4.5, as compared to AID’s native charge of +14 at neutral pH. This high net positive charge of AID is a unique feature amongst AID/APOBEC3s with known structure (−2, −6, +0.5, −9, and −3.5 of A3A, A3B-CTD, A3C, A3F-CTD, and A3G-CTD, respectively).

With this limitation, this structure nevertheless presents a unique opportunity for a comparison of structure determination methodologies. To this end, we compared the AID variant crystal structure with the computational–biochemical AID conformational ensemble (loop 2, 4, 6, and 8, denoted as loop 1, 3, 5, and 7, respectively, in other publications on APOBEC structures). Overall, the AID structures shared virtually the same tertiary structure and the variant structure confirmed some of the key secondary catalytic residues we posited would stabilize dC in the catalytic pocket (i.e., N51 and Y114). Most of the mutations in the AID variant were localized to L1, L2, and α1, regions, while α7 was deleted. There are also several notable differences: first, L2 adopts a more compact conformation relative to the core structure, likely due to the deletion of three residues in L2. We and others have previously shown that L2 plays a role in catalytic activity and AID:DNA binding of AID (58, 67). Second, L8 adopts a much more extended conformation in the AID variant. It was suggested this extended conformation stabilized larger purine bases upstream of the target cytidine, in contrast to other APOBECs whose shorter L8’s preferred pyrimidines upstream. However, the structure of L8 is stabilized by L2, which has been shown to modulate its compaction and substrate specificity (69). Additionally, the conformation of α7 relative to the surface of AID is uncertain, although some conformations place it in direct contact with L8 (58). Therefore, although L8 was not directly altered, mutation of L1-α1-L2 together with α7 deletion may indirectly perturb its conformation in the AID variant crystal structure.

Using our computational–biochemical approach, we also highlighted two DNA-binding grooves on the surface of AID, for both of which the positively charged R25 residues plays a major role in orienting the negatively charged DNA backbone (58). Recently, DNA-bound crystal structures of mutant A3A and an A3B-CTD chimera were shown to adopt a similar DNA-binding mode, wherein the DNA backbone was bound around the equivalent of R25 in AID (H29 and H212, in mutant A3A and A3B-CTD chimera, respectively) (57).

In this manner, X-ray and NMR structural elucidation of homologous APOBEC3s have provided direct physical support for notable features of AID observed using the computational–biochemical approach. These features include position and identity of catalytic residues, key DNA-contact residues as well as existence of occluded catalytic pockets, a novel regulatory mechanism.

## Conclusion

In summary, we draw the parallel to the Schrödinger’s Cat paradox that the catalytic pockets of AID/APOBEC3s appear to transition between dual states, one of which correlates with activity and the other with catalytic death, each with profound functional consequences. The second parallel between structure determination in the AID/APOBEC3 field and quantum physics is that X-ray crystallography and NMR determination of structures in the AID/APOBEC family have most often necessitated making extensive alterations to structures for and during the very act of observation. In contrast, the computational–biochemical approach used to solve AID’s functional structure relies on unobtrusive observation through prediction. Interventions are strictly reserved for the functional testing phase wherein structure predictions are rigorously scrutinized by conducting enzyme assays on a large library of variants including mutants, orthologs, and chimeras (Figure 1E). As described in the preceding section, it is important to note that this method is nonetheless dependent on the availability of multiple X-ray and NMR structure solutions, both in the beginning as templates and in the end, as independent methods to independently verify the key aspects of the structure.

In the future, as the relative contributions of each individual AID/APOBEC3 enzyme to immunity and cancer in different contexts become clearer, it will be important to test the hypothesis that catalytic pocket breathing differences among the AID/APOBEC3 family members impact their relative contributions, and to understand the extent to which this novel built-in safety switch is intertwined with other regulatory mechanisms, such as perhaps being modulated by aforementioned extrinsic binding partners, oligomerization or post-translational modifications. Although catalytic pocket closure has been described for other enzymes (131, 132), our discovery of such a functionally critical state in AID/APOBEC3s represents a novel regulatory mechanism for human DNA/RNA-damaging enzymes; hence, it is also important to ascertain how prevalent a regulatory mechanism dynamic catalytic pocket closure is in other DNA/RNA-editing enzymes, or whether it has evolved as a unique regulatory structural feature of the AID/APOBEC3 family.

## Author Contributions

JK (senior Ph.D. candidate) and ML (PI) both contributed to the writing of this article.

## Conflict of Interest Statement

The authors declare that the research was conducted in the absence of any commercial or financial relationships that could be construed as a potential conflict of interest.
